# Functionalization of Woven Fabrics with PBT Yarns

**DOI:** 10.3390/polym13020260

**Published:** 2021-01-14

**Authors:** Klara Kostajnšek, Krste Dimitrovski, Hüseyin Kadoğlu, Pinar Çelik, Güldemet Başal Bayraktar, Tuba Bedez Üte, Deniz Duran, Mustafa Ertekin, Andrej Demšar, Matejka Bizjak

**Affiliations:** 1 Department of Textiles, Graphics Art and Design, Faculty for Natural Sciences and Engineering, University of Ljubljana, Snežniška 5, 1000 Ljubljana, Slovenia; krste.dimitrovski@gmail.com (K.D.); andrej.demsar@ntf.uni-lj.si (A.D.); mateja.bizjak@ntf.uni-lj.si (M.B.); 2 Department of Textile Engineering, Faculty of Engineering, Ege University, Bornova, 35100 Izmir, Turkey; huseyin.kadoglu@ege.edu.tr (H.K.); pinar.celik@ege.edu.tr (P.Ç.); guldemet.basal@ege.edu.tr (G.B.B.); tuba.bedez@ege.edu.tr (T.B.Ü.); deniz.duran@ege.edu.tr (D.D.); mustafa.ertekin@ege.edu.tr (M.E.)

**Keywords:** PBT yarn, woven fabric, basic weave, terry, elasticity

## Abstract

Elasticity and recovery are important for clothing comfort, especially in the manufacture of apparel and sportswear. Recently, yarns containing PBT (polybutylene terephthalate), which are able to develop good elastic properties with high recovery after a finishing process (e.g., thermal treatment), have been used for this purpose. The aim of this work is to give a comprehensive overview of the use of PBT yarns in woven structure, with the aim of improving the elastic properties of cotton-like fabrics. The experimental part was divided into three main sequences to investigate the fabric properties (physical, elastic, UPF, comfort) influenced by (1) PBT-containing yarn structure, (2) weave and fabric structure (basic weaves and complex weaves) with PBT in weft direction, and (3) processing sequence—thermal treatment of PBT yarns or fabrics after weaving. According to the results, PBT-containing yarns have great potential for the production of lightweight elastic fabrics. The advantages of improving the elastic properties of fabrics by incorporating a relatively small amount of PBT yarns into the fabric only in certain areas, thereby minimally affecting the production costs, are demonstrated by a product with partially elastic areas obtained after thermal treatment.

## 1. Introduction

Elastic yarns have become indispensable for the textile industry, especially for apparel and sportswear production. Elasticity and elastic recovery are important for the clothing comfort or to meet other specific needs of other products. To achieve such properties, it is necessary to incorporate high elastic yarns into the fabric structure. Introducing Lycra [1,2,3,4] or other elastic spandex yarns in a relatively small quantity (2–5%) into the woven structure leads to the shrinkage of woven fabrics immediately after the weaving, resulting in additional stretching in one or both directions. Fabrics significantly change their properties, e.g., dimensions, mass per unit area, thickness, which results in the increasing some of their functional properties such as porosity, heat and air permeability, etc. [5].

The manufacturing process of elastic fabrics, especially the warping and weaving phases, is somewhat slower compared to the processing of the nonelastic ones, due to the excessive elasticity of elastic yarn. This leads to at least a decrease in production speed and in some cases also to quality problems. Recently, yarns containing PBT (polybutylene terephthalate) have been used for the same purposes with some improved effects [6].

The development of latent-elasticity fibers, which are capable of developing outstanding elastic properties with recovery after elongation exceeding 100% during a finishing process (e.g., dyeing), has created the opportunity for novel applications [7]. Due to characteristics these fibers are ideal for manufacturing highly elastic and comfortable garments. The elastic and elastic recovery properties of textured multifilament yarns made of these fibers provide greater freedom of motion and better comfort than less stretchable PA or PES/PET textured yarns.

**Figure 1:**
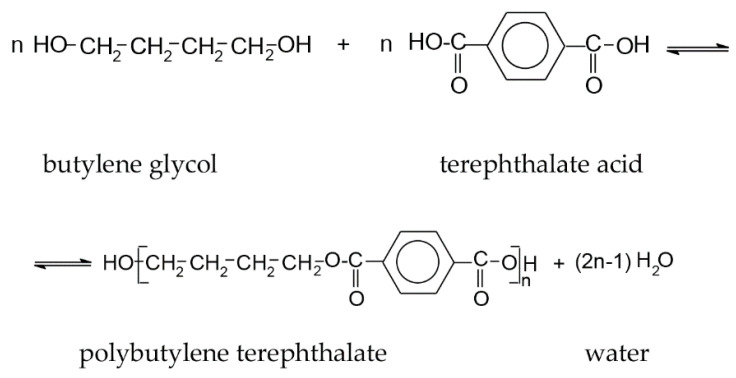
The chemical reaction for synthesis and structure of PBT polymer.

Polybutylene terephthalate (PBT) fibers are polyester fibers synthetized from terephthalic acid and 1,4 butanediol (butylene glycol). It has four methylene groups in structural formula that is two methylene groups more than the most often used polyester PET. The polymerization formula of PBT polymer is presented in Figure 1.

**Figure 2:**
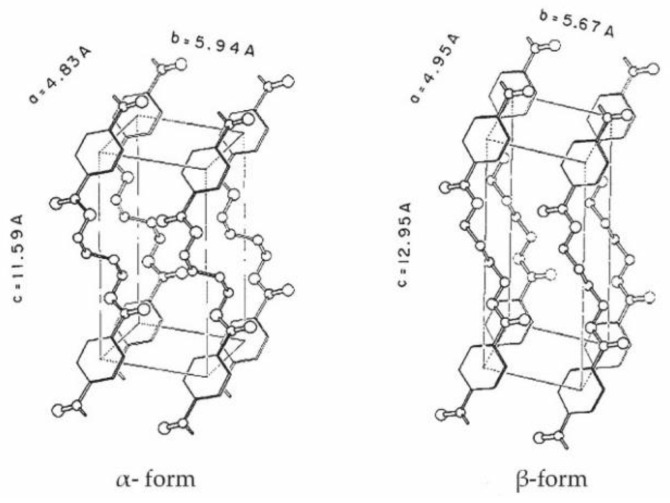
Two crystalline forms of the reversible molecular structure of PBT polymer elastic fiber: **left** α-form (after relaxation) and **right** β-form (under tension) [9].

The increased number of methylene groups in polymer molecules is important for flexibility of fibers. The resulting polymer possesses a high degree of crystallinity [8]. It is well known that the crystal form of PBT is reversely changed. The crystal structure of PBT transforms into β-form by the hot stretch treatment and into α-form by relaxation [8,9]. Both structures of the PBT crystalline form are given in Figure 2.

PBT fibers get their β-form during the process of friction texturing when they are under tension. In this case, the elastic properties of PBT yarns do not differ significantly from those of the PET fiber. This means that they can be processed in almost the same, well known and proven way as PET fibers. After PBT yarns have been incorporated into the fabric structure and subjected to thermal treatment (e.g., during dyeing or thermofixation process), PBT yarns change to their α-form, which causes shrinkage of the fabric depending on the amount of PBT yarn and fabric structure. This shrinkage actually represents the part of the increased elasticity of the fabric that contributes to the behavior of the fabric and increases its usefulness, especially in terms of comfort [5,6,10,11].

PBT absorbs only a small amount of water, therefore, is highly resistant to perspiration and humidity and dries very quickly. Longer exposure of PBT to hot water or steam leads to depolymerization of molecules. Due to high crystallinity, PBT has excellent chemical resistance to most organic solvents and it is also wear resistant [7]. PBT fibers have good color-fastness, chlorine resistance, UV resistance, and light-fastness similar to that of polyester and can be dyed at high temperatures. This high elastic yarn with good stretch and recovery properties is both dimensionally stable and soft [10].

All these features distinguish PBT, the elastic but nonelastomeric yarn, as suitable for use in knitted fabrics [12,13] and woven fabrics [5,6,14,15,16], for blending with all natural or synthetic yarns in various yarn structure forms (filament, core-spun yarn, dual-core yarn) [14,16,17,18]. PBT is therefore particularly suitable for sportswear, underwear and outerwear, and also for textiles in car interiors or for floor coverings. Comfort clothing containing PBT in its structure is characterized by a pleasant feel as well as body shaping and hugging capability and it is successfully used despite higher PBT prices than those of PES yarns.

PBT is not only used in the textile industry, but also in the automotive, electrical, and consumer applications. Due to some excellent polymer properties already mentioned (dimensional stability, good electrical and mechanical properties) and its good processability (low melting point, fast crystallization on cooling, and excellent flow behavior in the molten state) it is very often modified with other polymers and particulate filler in nonwoven forms. PBT is suitable for injection molding or extrusion processes. For this reason, thermoplastic PBT composites [19,20,21] or PBT fiber composites (for capacitive touch panels, smartphones) [22] are often used in technical fields. In the medical field, PBT nonwovens are used for blood filtration, as carriers, absorbents [23,24,25], and also as technical cartilage tissue based on the used PBT fibers [26].

The purpose of this paper is to provide a comprehensive overview of research into the use of PBT yarns, with the aim of improving the elastic properties of woven fabrics in a way that differs from the established use of elastic yarns.

The main factors influencing the presence of PBT yarns in the woven structure are discussed: the fineness of the warp/weft yarns, their densities, and especially weaves depending on the amount of PBT added, the type of PBT-containing yarn, and the positioning of PBT yarns in the weave structure. The overall study had the primary objective to find out by how much the stretchability of the manufactured fabrics improves after thermal treatment and how other properties are affected. The research was divided into three thematic sections according to the preliminary investigations, with which we tried to define a specific framework for further experiments [6,15,16,27].

### 1.1. The Influence of PBT Yarn Type on the Weaving Process

Three types of PBT containing yarns were studied: PBT filament, core-spun yarns with a PBT core surrounded by cotton or CV (viscose), and folded PBT filaments with other yarn types.

When PBT filament has been combined with thicker yarns as weft in weaving in certain sequences, the cross marks/lines have appeared in the fabrics after thermal treatment, except when used in combination with similar types of synthetic fibers. When PBT filament has been combined with each weft, the shrinkage of the fabric after thermal treatment was too high (high elasticity). An additional drawback is that the breaking strength of PBT was reduced after treatment, resulting in deterioration of the mechanical properties of the fabric. When PBT is used in folded yarn in combination with natural yarns, at least the folding preparation stage is required. When folded PBT yarn was used as each weft, the shrinkage of the fabric after treatment was too high (high elasticity).

Using PBT as core surrounded by other fibers in a core-spun yarn has proven to have the least disadvantages: no marks/lines on the fabric surface after treatment; easy dyeing; can be used in sequences as weft and thus the degree of elasticity can be planned/controlled. The only disadvantage is the higher price of the yarn. The production price of the fabric increases, nevertheless, the percentage of PBT yarn in the fabric is as low as possible. The use of PBT yarns does not cause any problems in the weaving process, as the elasticity of yarns is gained after thermal treatment.

### 1.2. The Influence of Fabric Construction on the Degree of Shrinkage

The fabric construction must be loose enough, with enough floating threads to allow shrinkage after thermal treatment. Here, the weave plays an important role. The more threads float (fewer interlacing points), the more the fabric shrinks after thermal treatment. Yarns with PBT can be inserted in one direction only, preferably in the weft, to obtain the desired elastic properties, but they can also be inserted in sequences.

### 1.3. The Influence of the Thermal Treatment Method on the Uniformity of Shrinkage

During the ironing process, the fabric was exposed to steam, which caused the fabric to shrink, but the degree and uniformity of shrinkage could not be controlled.

When the fabric was treated in boiled, distilled water for half an hour, the results were more predictable, and the shrinkage of the fabric was uniform across the width. The method is more practical in production environments as it can be used in the washing or desizing process.

## 2. Materials and Methods

### 2.1. Overview of Experimental Work

The experimental part was divided into three main sequences to investigate different influences:

- Influence of elastic yarn structure on the properties of fabrics;
- Influence of fabric structure, with PBT weft, on the properties of fabrics;
- Comparison of properties of fabrics woven with thermally pretreated PBT yarns with the those of fabrics woven with untreated PBT yarns and thermal treatment after weaving.

#### 2.1.1. Influence of the Elastic Yarn Structure on the Properties of Fabrics

To investigate the impact of structure of PBT yarn and compare them with other elastic yarns, four different core filaments were used for production of core yarns surrounded with cotton sheets. PBT (7.8 tex) and elastane (7.8 tex) cores were fed in three different tensions (1:3; 1:3.5, 1:4). All core spun yarns (30 tex) were produced with the same twist coefficient on the lab ring frame (Pinter, Merlin Caipo S.A.U., Barcelona, Spain). Six fabric samples were woven on Sulzer rapier weaving machine, with the same 100% cotton warp (59 tex) and different wefts (PBT/Co core yarn, Elastane/Co core yarn, PBT filament: cotton yarn in sequence 1:1 and 100% cotton yarn). All samples were woven with the same on-loom settings: 12 ends/cm, 28 picks/cm, and in twill 1/3 Z. Woven fabrics containing PBT yarns were treated in boiling water for 30 min to impart elasticity [16]. The fabric characteristics are given in Appendix A, Table A1.

In the study [16] the following properties of woven samples were investigated: physical properties (mass per unit area SIST EN 12127:1999, thickness of textiles SIST EN ISO 5084:1999), the woven fabric resistance to stretch (TSE-TS 6071), air permeability (EN ISO 9237:1995), thermal and water-vapor resistance under steady-state conditions ((Alambeta, Sensora Co., Liberec, Czech Republic), ISO 11092:2014), dimensional stability after thermal treatment (ISO 3759:2011). Results of resistance to stretch, dimensional stability and thermal comfort properties, weight, and thickness values of the fabric samples are given in Appendix A, Table A2 and Table A3.

Another feature worthy of discussion when using PBT yarns for light summer clothing is UV protection. In the study [27], the influence of Lycra and PBT in woven structure on UV properties of fabrics is discussed. The fabric samples were produced under the same settings as in the study mentioned above [16], with the exception that all weft yarns (PBT/Co core yarn, Elastane/Co core yarn, PBT filament: cotton yarn in sequence 1:1 and 100% cotton yarn) were thinner, namely 24.5 tex.

After weaving, the physical properties of the fabrics and the UV properties were measured. Subsequently, all samples were thermally treated in boiled distilled water for 30 min. After the treatment, the physical and UV properties of the fabrics were measured again. The fabric weight and thickness were measured in accordance with SIST EN 12127:1999 and SIST EN ISO 5084:1996, respectively. UV transmission and reflection were measured according to the standard EN 13758-1:2001 on the UV-visible spectrophotometer Lambda 800, equipped with PELA-1000 (PerkinElmer, Inc., Waltham, MA, USA). Results of the selected physical properties and the UV properties of examined samples are given in Appendix A, Table A4 and Table A5.

#### 2.1.2. Influence of Fabric Structure, with PBT Weft, on the Properties of Fabrics

We continued with an investigation of the influence of different yarn densities, the sequence of insertion of PBT yarn in the weft direction and different weaves on the properties of fabrics with PBT yarn.

**Figure 3:**
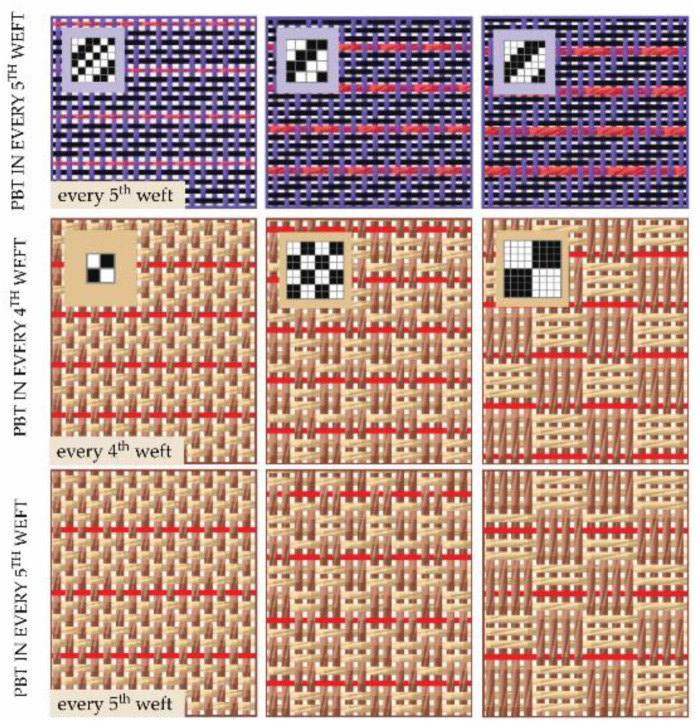
Simulations of woven fabrics shows the weave, linear density of the PBT yarns, and different weft sequence used.

**Figure 4:**
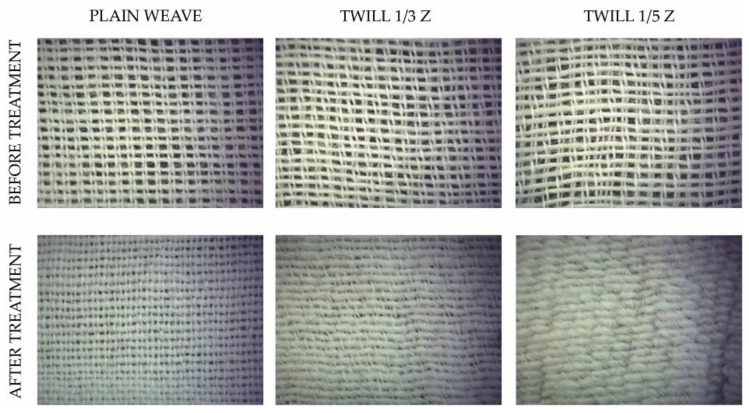
Pictures of samples produced with untreated Co/PBT yarn in weft, before and after thermal treatment of fabrics.

Nine samples of fabrics, differing in the structure of the PBT-containing yarn, the weave and sequence of PBT in the weft, were all produced on the laboratory rapier weaving machine (Minifaber, Seriate (BG), Italy), with the warp of 100% cotton (8 × 2 tex) at on-loom density of 20 ends/cm. Three of them, containing PBT in every fifth weft (sequence: four cotton yarns, one yarn containing PBT), were designed and produced in three different twill weaves, (rows 2, 3, and 4 in Table 1, Figure 3). By using different twill weaves, we also wanted to evaluate the influence of float length on fabric properties before and after thermal treatment. Figure 4, first row, shows three weaves used and simulations of woven fabrics [28] with well depicted weft sequence.

We also studied the changes in elastic properties of woven fabrics and cotton series with the addition of two different sequences of viscose/PBT yarn in the weft direction and different weaves to achieve better elastic properties. Viscose/PBT core yarn was 10 × 2 tex (50/2 Nm) with PBT 5.6 tex in the core, where the PBT contributes 28% of yarn mass. Six samples were designed and produced, with different sequences of viscose/PBT combinations in every fourth and fifth weft and in three different weaves (rows 5, 6, and 7 rows in Table 1, Figure 3). After weaving, all fabrics were thermally treated—boiled in distilled water for 30 min and dried for 24 h in a state of free tension [5,17].

In this study, the following properties of all woven samples were investigated: physical properties (mass per unit area SIST EN 12127:1999, number of threads per unit length SIST EN 1049-2:1993, thickness of textiles SIST EN ISO 5084:1996), tensile properties of fabrics (SIST EN ISO 13934-1), air permeability (ISO 9237:1995), and for samples from 4 to 6 (Table 1) the abrasion resistance by Martindale method (SIST EN ISO 12945-2) was also measured. The results of shrinkage after thermal treatment and physical properties of samples are shown in Appendix B Table A6 and Table A7.

The study was further extended by investigating the influence of the use of PBT on the properties of complex fabrics, such as terry fabrics. The range of applications of terry fabrics with increased elasticity is much wider than the classical one. They would also be suitable for sportswear and children’s clothing, blankets, bedding, technical applications or as innovative products (universal fitted sheet/bedding/towel for inflatable pillows).

In this work the influences of different constructions of the fabrics on the elastic and other usage properties of terry were investigated. To study the influence of constructional parameters on terry properties, six different terry samples were used, which were industrially produced on terry weaving machine (Vamatex, Deinze, Belgium). They differed in warp yarns (OE cotton yarn, 74 tex and carded ring spun yarn, 60 tex) and weft yarns (OE cotton yarn, two core spun yarns with cotton sheet—one with PBT core and the other with Lycra core, PBT multifilament), as far as the pile yarn was the same for all samples (OE cotton, 37 tex). The face-side three-pick terry, with base weave mixed rip 2/3, and the same warp sequence were used for all six terry fabrics, but last two terry fabrics had different weft sequences, as shown in Table 2.

After thermal treatment, boiling in water for 30 min to impart elasticity, basic properties of treated and untreated terry fabrics were investigated: crimp of yarn in fabric (ISO 7211-3:1984), mass per unit area (SIST EN 12127:1999), tensile properties of fabrics (SIST EN ISO 13934-1:2013), bursting properties of fabrics (ISO 13938-2:1999), vertical wicking (measures the distance in mm at a given time; (AATCC Test Method 197), dimensional changes of treated samples (ISO 3759:2011). The results of physical mechanical properties of the samples before (b.t.) and after (a.t.) treatment are shown in Table 3.

#### 2.1.3. Comparison of Properties of Fabrics Woven with Thermally Pretreated PBT Yarns with the Those of Fabrics Woven with Untreated PBT Yarns and Thermal Treatment after Weaving

Previous investigations of elastic fabrics containing PBT were performed on treated fabrics in boiling water for 30 min, which gave the fabrics elastic properties. From the weaving point of view, this is an ideal solution, as the weaving process is undemanding compared to weaving with elastic yarns. The properties of the finished Co/PBT fabrics are highly dependent on the design parameters [10,16,17].

During the treatment process, PBT filaments gain elastic properties, while at the same time the cotton fibers shrink and swell. The shrinkage of Co/PBT yarns in the loose state differs from the shrinkage in the clamped state (i.e., yarns in fabric structure), which is influenced by many factors, especially the weave and density. A larger number of interlacing points in the structure means more friction surfaces that inhibit the contraction of fibers/threads in the fabric.

The study shows that the elasticity of Co/PBT fabrics after thermal treatment depends not only on the earlier mentioned fabric settings (percentage of PBT in the yarn; yarn structure and fabric settings), but also on the settings on the weaving machine (yarn tension, speed, weft insertion technology, etc.). By producing fabric samples from treated and untreated Co/PBT yarns with the same production parameters, we attempted to demonstrate the influence of the fabric setting and the sequence of the treatment process on the fabric properties [14].

For the study [14], six fabric samples were produced on Minifaber laboratory rapier weaving machine, with the 100% cotton (8 × 2 tex) warp and Co/PBT core yarn (40 tex) with PBT core 8.3 tex as weft. The on-loom density of the warp was 20 ends/cm and density of the weft was 15 picks/cm. Three of them, made in three different weaves (plain, twill 1/3 Z, and twill 1/5 Z) contained untreated Co/PBT weft and after weaving the samples were treated in boiling water to impart elasticity. The other three fabrics were also woven using the same three weaves, except that the Co/PBT weft yarn was previously treated in boiling water and then samples were woven. Fabrics characteristics are shown in Appendix C, Table A8.

After weaving and thermal treatment, basic properties of yarns and woven samples were investigated: dimensional change (shrinkage) of Co/PBT yarn after treatment, physical and constructional properties (number of threads per unit length SIST EN 1049-2:1993, crimp of yarn in fabric ISO 7211-3:1984, mass per unit area SIST EN 12127:1999, thickness of textiles SIST EN ISO 5084:1996), dimensional changes of treated samples (ISO 3759:2011), air permeability (EN ISO 9237:1995). Dimensional changes (shrinkage) of the samples in warp and weft direction were evaluated according to the standard before and after thermal treatment. Dimensional changes of Co/PBT yarn after treatment were evaluated by measuring the yarn length before and after treatment in boiling water. During the 30-min treatment in boiling water in a loose state, the Co/PBT yarn shrank by 36.5%, resulting in a change in yarn count. The results of tested properties are shown in Appendix C, Table A9.

Images of samples woven with thermally untreated Co/PBT yarn in weft, before and after thermal treatment of fabrics, were taken with the stereomicroscope 65.560 NOVEX using the digital camera CMEX 5000, magnification 6.5 × 0.65 (Figure 4).

Based on previous research, the main objective of the entire study was to find the most appropriate implementation in a final product by incorporating potentially elastic yarns into fabrics only in specific areas of the fabric where elasticity is required in the final product [15], i.e., not throughout the entire fabric. Fitted sheets and related products are an example of such a widespread application, where an inelastic top sheet and the elastic edges that form the bottom of the sheets are required. The elastic areas of the sheets take the role of the elastic strips/cords, that are normally sewn into the edges of fitted bed sheets.

The technology allows the insertion of potentially elastic threads in only one direction or in two directions. For technological reasons, it is easier and in most cases sufficient to insert potentially elastic threads in only one, namely the weft direction. After cutting and sewing, the final product made of raw, thermally untreated fabric must be thermally treated (e.g., by exposure to steam during ironing in production) to obtain the shape of the final product [15].

## 3. Results and Discussion

In this section only the most important influencing factors, according to the division of experimental part, are discussed.

### 3.1. Influence of Elastic Yarn Structure on the Properties of Fabrics

**Figure 5:**
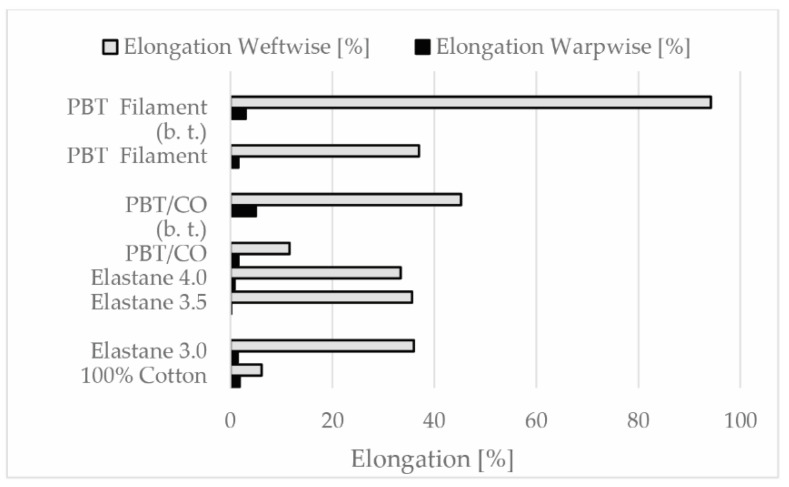
Elongation values (warp and weftwise) of fabric samples ((b.t.)—before thermal treatment).

The results showed that [16] thermal treatment (washing) had a significant effect on the elongation and permanent elongation values of the fabric samples containing PBT. After washing in boiling water, the elongation value in the weft direction increased by 33.6% for Co/PBT and 57.2% for the PBT filament fabric sample. PBT filament and Co/PBT fabric samples showed higher elongation values in both directions compared to the other fabric samples (Figure 5).

The shrinkage value of the PBT filament fabric sample and the fabric samples with elastane yarns was about 35% in the weft direction. In terms of elastic properties, the PBT filament and Co/PBT fabric samples showed higher elongation values in both directions. PBT filament fabric sample with higher elongation and similar shrinkage properties offered more elastic behavior with lower weight and thickness values.

The fabric samples with elastane yarns had lower air permeability compared to the others. The 100% cotton fabric sample had the highest air permeability, followed by PBT filament and PBT/cotton fabric samples.

PBT filament fabric samples and fabric samples with elastane yarns (elastane 3.0, 3.5, 4.0) had the highest and lowest water vapor permeability values, respectively.

The fabric samples with elastane yarns had higher thermal conductivity and gave a cooler feeling on first touch due to their higher heat absorption properties. The fabric sample with PBT filaments had lower thermal conductivity and absorption capacity values.

**Figure 6:**
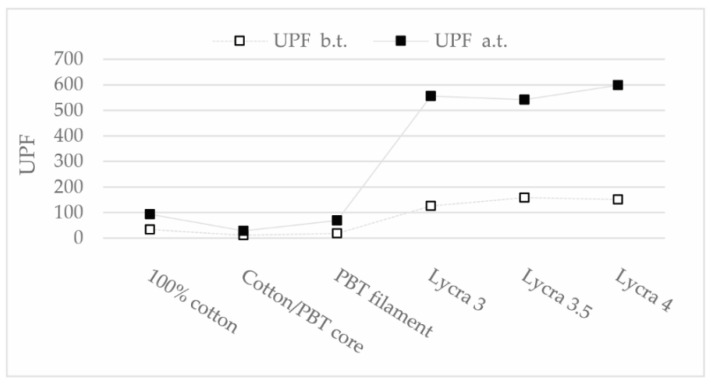
UPF of samples before (b.t.) and after (a.t.) treatment.

When Lycra was used, shrinkage was present immediately after weaving and the samples immediately provided excellent UV protection. Fabrics containing PBT prior to thermal treatment did not have adequate UPF, however the thermal treatment caused additional stretching of the samples, resulting in a tighter structure, an increase in mass per square meter and increased thickness. As a result of the tighter structure and greater mass as barrier to UV light penetration, the samples with PBT filament obtained a UPF greater than 50 (Figure 6) [27].

Raw fabrics containing Lycra in the weft stretched to an areal density of 310 g/m^2^ immediately after weaving, as expected, and had a UPF of more than 120 (126–157), proving that the fabrics have a completely closed structure after stretching, which does not allow direct penetration of UV light. There is no significant difference between samples with 3, 3.5, or 4 times extended Lycra in yarn structures. Fabrics made of cotton and fabrics containing PBT possessed very little stretchability after weaving and did not receive sufficient UPF, partly because their structure is not completely closed before thermal treatment, allowing direct penetration of UV light.

Thermal treatment causes additional stretching of the samples, further increasing the mass per square meter and the thickness of all samples. For the Lycra-containing fabrics, the mass per square meter increased by a third, and for the cotton fabrics and the samples with PBT multifilament by about a quarter, so that they all reached at least 226 g/m^2^. Taking into account the mass per square meter as a barrier to UV light penetration and the additional closure of the fabric structures, the UPF of the Lycra-containing fabrics increased to over 400. The cotton fabric and the fabric with PBT filament obtained an excellent UPF of 60 and 50, respectively. Only the sample with cotton/PBT core yarn in the weft obtained an inadequate UPF of only 17 (Figure 6).

If the fabric construction is closed enough, a similar effect can be achieved with a lower fabric weight and colored yarns in the right colors [29,30].

The use of Lycra and PBT in the fabric construction contributes to the closure of the fabric structure in both cases; only Lycra does this more intensively and immediately after weaving, PBT after thermal treatment. However, the decision of which material to use also depends on other desirable physical, mechanical, and permeability properties of fabrics, taking into account the simplicity of manufacture (ease of production), maintenance, durability, and price.

According to the results, PBT filament yarns have a great potential for the production of lightweight elastic fabrics. Not to mention the fact that PBT has better resistance to finishing processes and better durability than elastane.

### 3.2. Influence of the Fabric Structure, with PBT Weftwise, on the Properties of Fabrics

The use of different types of PBT yarns as weft in woven structures can improve the elastic properties of woven fabrics in the direction of insertion to a level similar to that of knitted fabrics. In addition, most other physical and mechanical properties remain approximately the same or deteriorate only slightly. The degree of improvement depends on the type of PBT-containing yarns and their properties, as well as the construction parameters of the fabric. In the study, the lowest results in weft elasticity were obtained with Co/PBT core yarn. The results obtained with PBT multifilament and CV/PBT core yarns were similar and among the best in fabrics with float lengths above 2 and 3 threads.

**Figure 7:**
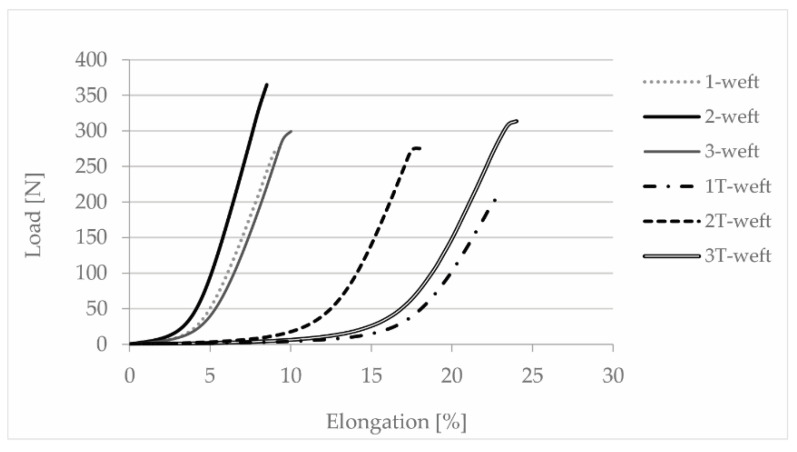
Load/elongation curves of woven samples in weft direction before and after treatment.

The elongation at break of the fabric samples in the weft direction after thermal treatment increased dramatically and almost reached the level of knitted fabrics. It can be seen that the fabrics obtained an elongation of about 15% under the load of only 10–20 N, which is three times that of the untreated fabrics (Figure 7).

**Figure 8:**
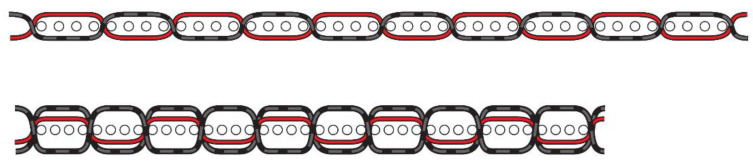
Model for the change in structural properties of fabrics with introduced PBT-containing yarns in the weft after thermal treatment (red lines show PBT-containing yarns).

At the same time, most other physical and tensile properties remained without significant changes or even improved. The model of the changing structural properties given in Figure 8 shows how the fabrics shrank after treatment by the PBT-containing yarn, giving them significantly greater mass and thickness, better thermal insulation properties, and greater fabric softness.

As a result of the shrinkage in width, the density of the warp yarns increased and so did the tensile strength of the fabric in the warp direction. The only significant decrease occurred in air permeability, which decreased by about 20% due to the decrease in pore size in the fabric structure.

As a general conclusion, which included studies with the addition of two different frequencies of viscose/PBT yarns in the weft direction and different weaves, we can say that the use of a relatively small amount of PBT in the weft can improve the elastic properties of fabrics, especially in the weft direction, without ruining or even improving other properties. We also found that using PBT in every 4th or 5th weft does not play an important role in changing the physical and mechanical properties, so it makes more sense to use PBT in every 5th weft for economic reasons.

In general, we get satisfactory results by using PBT yarn in the weft, and it is possible to get better improvements in physical and mechanical properties of fabrics by combining different PBT yarns in different warp and weft yarns.

Since PBT-containing yarns develop their latent elasticity after thermal treatment, they can also be used in warp direction without causing significant problems in weaving preparation and weaving. This makes them ideal for the production of so-called stretch fabrics.

The decision to extend the research to the use of yarns containing PBT in compact structures such as woven terry cloth was based on the fact that the elasticity and improved quality of terry fabric lead to completely new applications. An important factor for the quality of terry fabrics, especially the absorbency, is the weight ratio between pile and ground fabric. The higher loop ratio can be achieved in two ways: by higher loops and by higher loop density. However, there are technological limitations due to weaving characteristics and special weaving technique. Another technological challenge is the weaving of terry with elastic weft (with the addition of Lycra). The use of Co/PBT core yarns with potential elasticity in the weft should enable an improvement of the fabric parameters. The technological limit for achieving a higher loop density in terry fabrics is theoretically exceeded after thermal treatment, when the elastic weft causes the shrinkage of the fabric, thus increasing the number of loops per unit area.

**Figure 9:**
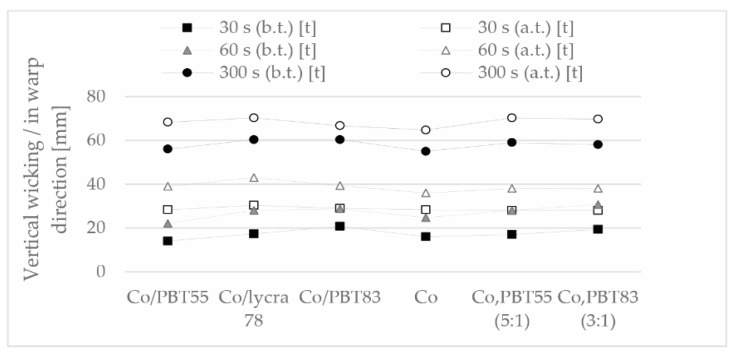
Moisture management (vertical wicking) of the samples in warp direction (measures distance in mm at a certain time), before (b.t.) and after (a.t.) thermal treatment.

**Figure 10:**
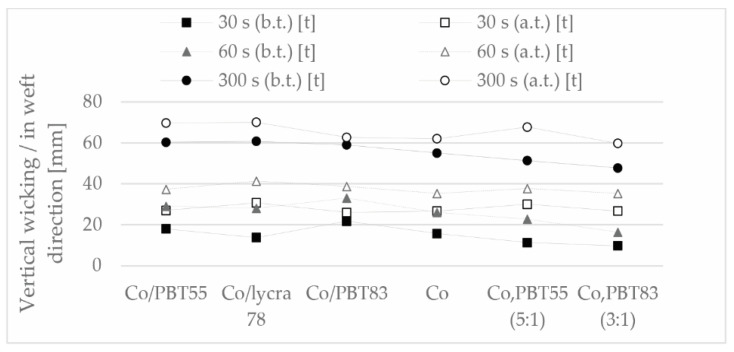
Moisture management (vertical wicking) of samples in the weft direction (measures distance in mm at a certain time), before (b.t.) and after (a.t.) thermal treatment.

The moisture transport of terry fabrics depends mainly on the capillary capacity and the moisture absorption capacity of the fibers. These properties are particularly important for terry fabrics, bathrobes, or sportswear, which is why it is necessary to determine the wicking behavior of the samples. The absorption properties of the samples and the change in absorption after thermal treatment in the warp direction are shown in Figure 9 and in the weft direction in Figure 10.

The increase in moisture absorption capacity after thermal treatment was not only due to the increase in loop density, but also to the removal of starch residues from the yarn during the thermal treatment phase in boiling water. The vertical wicking effect was similar for all samples after thermal treatment in the same time interval, this phenomenon was observed in all three time intervals. As expected, the Lycra sample with the highest elasticity and thus the highest loop density also had the best wicking.

During the investigation, influences of the type of yarn containing PBT (PBT multifilament and Co/PBT core yarn) on the elastic and other properties of the terry fabric were considered. When PBT filament was combined with thicker yarns as weft in certain sequences during weaving, the transverse marks/lines appear in the terry fabric, which was found to be a nonoptimal solution for obtaining a more elastic terry fabric due to the stripes and difficulties in the weaving process. The use of Co/PBT core yarn proved to be the solution with the least disadvantages: no marks/lines on the fabric surface after thermal treatment; can be used in sequences as weft and thus the degree of elasticity can be planned/controlled.

The mass per unit area of all samples increased after thermal treatment due to shrinkage, but it can be seen from the results that they behave differently from the fabrics in conventional weaves due to the compact terry weave. The greatest shrinkage was shown by the terry sample with Lycra in the weft, which already causes shrinkage during weaving, so this sample has satisfactory elasticity. All other mechanical properties of all samples improved after heat treatment due to shrinkage and higher thread density.

The study of how woven structure affects the improvement of elastic properties can be concluded with knowledge that fabric construction must be loose enough, with enough floating threads to allow shrinkage after thermal treatment, and here the weave plays an important role. The more threads float (fewer interlacing points, less friction), the more the fabric shrinks after thermal treatment. The terry weave is compact and designed to prevent the loops from being pulled out, so the degree of elasticity achieved by terry fabrics is lower compared to conventional fabrics.

### 3.3. Comparison of the Properties of Fabrics Woven with Thermally Pretreated PBT Yarns with the Properties of Fabrics Woven with Untreated PBT Yarns and Thermally Treated after Weaving

The investigation showed a large difference between samples with pretreated Co/PBT yarns in the weft direction and samples with Co/PBT core yarns treated after weaving.

**Figure 11:**
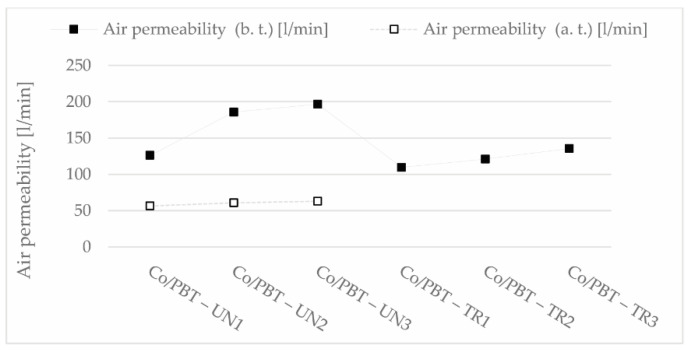
Air permeability of the samples before (b.t.) and after (a.t.) treatment.

The air permeability values of samples with untreated Co/PBT yarns (designation UN1 to UN3 in Figure 11) were reduced by more than 50% after treatment, while these values of samples with pretreated Co/PBT weft yarns (designation TR1 to TR3) showed better air permeability properties. The results showed that the treatment of Co/PBT samples after weaving significantly reduced air permeability (Figure 4 and Figure 11), as the fabrics with longer floats had a very dense surface (Figure 4). The study showed a large difference between samples woven with pretreated Co/PBT core yarns in the weft direction and samples woven with Co/PBT core yarns treated after weaving.

The air permeability values of samples with untreated Co/PBT yarns (designated UN 1 to UN3 in Figure 11) were reduced by more than 50% after treatment, while these values of samples with pretreated Co/PBT weft yarns (designated TR1 to TR3 in Figure 11) showed better air permeability properties. The results showed that the treatment of Co/PBT samples after weaving significantly reduced the air permeability (Figure 4 and Figure 11), as the fabrics with longer floats had a very dense surface (Figure 4).

Samples with Co/PBT core yarn in the weft direction treated after weaving had higher weight and thickness, these values increased as a function of weave (length of floating yarns and thus yarn contraction ability).

After treatment, the yarn densities of the Co/PBT-UN samples were increased, resulting in higher warp and weft shrinkage, it being evident that the samples with longer floats and higher warp density had a higher percentage of crimp in the weft direction. The 3D fabric surface obtained by treating and thus shrinking the Co/PBT-UN samples resembled crepe fabrics and provided a soft feel to the fabric. However, two disadvantages resulted, namely reduced air permeability values and excessive dimensional changes in terms of fabric width change.

## 4. Conclusions

The conclusions summarize the findings of the whole study, highlighting all the benefits of using PBT as an alternative solution to increase the elasticity of fabrics, and show the potential of implementing the research findings into new functional products, such as fitted sheets.

**Figure 12:**
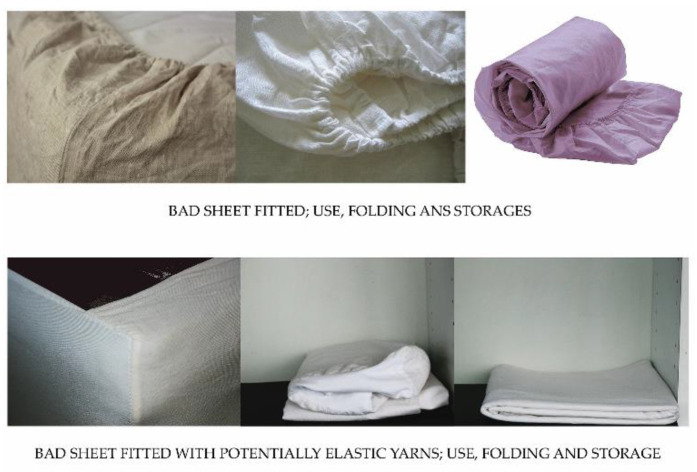
The differences in the use, folding, and storage between two types of bed sheets.

The benefits of improving the elastic properties of fabrics by incorporating potentially elastic yarns into the fabric only in specific areas, thus minimally affecting production costs, are demonstrated by comparing bed sheets with partially elastic areas to commercially available fitted sheets:

- Lower fabric consumption and easier sewing, as there is no need to sew elastic into the edges, resulting in lower sewing costs.
- Faster and easier drying of the product, especially at the edges where conventional fitted sheets have several layers of folded fabrics sewn together, including elastic strips/cords.
- Easier folding and storage (smaller volume) (Figure 12).
- Almost perfect fit to the bed dimensions (Figure 12).
- Another advantage is the easy ironability even on ironing machines in laundries. This is an important factor for customers such as hospitals, nursing homes, hotels and restaurants, etc., where the cost of maintaining the bed linen is of great importance.

Our study shows that basic research is a necessary but not yet sufficient factor for the improvement of the existing product or the development of a new product intended for commercialization. After the findings of basic research, applied research is necessary, which takes into account the optimization of technological, production, human, economic, and marketing factors. Ultimately, any newly developed product must face competition from products already on the market. It must have a special function and feature or have a better design, all at the same or even lower price than the competing products. Meeting all these requirements to be successful in the market is almost impossible. A sufficient criterion is usually linked to the requirement that the product has an added value for the user and at the same time is profitable for the manufacturer and retailer.

## Figures and Tables

**Table 1:** Characteristics of samples to evaluate the effects of different float lengths and weft sequences.

Designation	PBT Weft Characteristics	Weave	On-Loom Settings
1 _TWILL_	PBT Filament 7.8 tex	Twill 1/1/2/2, Twill 2/2, Twill 3/3	Warp, weft: 100% Co, 8 × 2 tex Warp density = 20 ends/cm Weft density = 25 picks/cm PBT in every 5th weft
2 _TWILL_	Cotton/PBT core yarn (30 tex) with PBT core 7.8 tex		
3 _TWILL_	CV/PBT core yarn (20 × 2 tex), with PBT core 5.6 tex		
4 _PLAIN_	CV/PBT core yarn (10 × 2 tex), with PBT core 5.6 tex	Plain Basket 4/4 Basket 8/8	Warp, weft: 100% Co, 8 × 2 tex Warp density = 20 ends/cm Weft density = 20 picks/cm CV/PBT in every 4th and every 5th weft
5 _BASKET_			
6 _BASKET_			

**Table 2:** Basic constructional parameters of face-side three-pick terry (base weave: mixed rip 2/3).

Sample Designation	Base Warp	Pile Warp	Weft	Warp and Weft Sequence
Co/PBT55	OE cotton, 74 tex	OE cotton, 37 tex	Co/PBT core yarn (39 tex) with 5.5 tex PBT core	Warp sequence: 2 base: 2 pile Weft sequence: 1 Co/PBT
Co/lycra8	OE cotton, 74 tex		Co/lycra core yarn (39 tex) with 7.8 tex lycra core	Warp sequence: 2 base: 2 pile Weft sequence: 1 Co/PBT
Co/PBT 83	OE cotton, 74 tex		Co/PBT core yarn (39 tex) with 8.3 tex PBT core	Warp sequence: 2 base: 2 pile Weft sequence: 1 Co/PBT
Co	OE cotton, 74 tex		OE cotton (39 tex) (reference sample)	Warp sequence: 2 base: 2 pile Weft sequence: 1 Co/PBT
Co,PBT55 (5:1)	Ring spun cotton, 60 tex		OE cotton (39 tex), PBT multifilament (5.5 tex)	Warp sequence: 2 base: 2 pile Weft sequence: 5 Co:1 PBT
Co,PBT83 (3:1)	Ring spun cotton, 60 tex		OE cotton (39 tex), PBT multifilament (8.3 tex)	Warp sequence: 2 base: 2 pile Weft sequence: 3 Co: 1 PBT

**Table 3:** Properties of terry samples before (b.t.) and after (a.t.) treatment.

Sample		Mass (g/m^2^)	Crimp (%)			Dimensional Stability (%)		Breaking Force (N)		Elongation (%)		Bursting Strength (daN/cm^2^)
			Ground Warp	Pile Warp	Weft	Warp	Weft	Warp	Weft	Warp	Weft	
Co/ PBT55	b.t.	468.1	7.2	413.2	22.6	/	/	398.1	408.6	14.4	38.0	2.08
	a.t.	522.0	12.0	463.0	22.6	−2.8	−0.6	419.3	458.2	19.1	40.8	2.67
Co/ lycra 78	b.t.	709.3	16.4	457.6	67.8	/	/	663.8	311.7	23.5	90.2	2.70
	a.t.	764.1	16.2	462.4	70.4	−3.2	−0.9	637.6	332.7	26.7	110.0	3.04
Co/PBT83	b.t.	509.3	9.4	460.4	20.6	/	/	448.8	443.8	17.2	38.7	2.48
	a.t.	520.7	12.2	462.2	21.8	−1.2	−1.8	457.5	397.0	18.7	40.8	2.69
Co	b.t.	501.3	10.0	517.6	17.0	/	/	375.7	422.3	14.1	24.5	2.22
	a.t.	524.1	11.6	553.4	15.0	−3.4	1.4	415.1	471.5	19.8	23.7	2.70
Co,PBT55 (5:1)	b.t.	486.6	8.4	491.6	16.0	/	/	382.9	355.1	14.5	26.7	2.14
	a.t.	515.8	12.0	522.6	18.0	−3.6	0.6	393.6	406.4	19.4	27.2	2.61
Co,PBT83 (3:1)	b.t.	372.5	8.6	284.0	18.8	/	/	446.1	302.3	16.3	29.01	2.75
	a.t.	390.2	12.0	296.2	18.8	−2.6	−0.2	463.0	313.4	19.4	29.5	2.72

## Data Availability

The data presented in this study are available on request from the corresponding author.

## Appendix A

**Table A1:** Properties of terry samples before (b.t.) and after (a.t.) treatment.

Designation	Weft Characteristics	On-Loom Settings
PBT/CO	PBT core yarn	Warp density = 12 ends/cm Weft density = 28 picks/cm Weave: twill 1/3 Z
Elastane 3.0	Elastane core yarn with different tension	
Elastane 3.5		
Elastane 4.0		
PBT Filament	Weft sequence 1:1 (1 cotton weft: 1 PBT filament)	
100% cotton	100% cotton yarn	

**Table A2:** The elongation and dimensional stability values of the fabric samples.

Designation	Direction of Measurement	Elongation (%)	Permanent Elongation (%)	Dimensional Stability (%)
100% Cotton	Weftwise	6.13	0.4	−16.38
	Warpwise	1.8	0.2	−5.81
Elastane 3.0	Weftwise	36	−3	−34.86
	Warpwise	1.4	−0.6	−6.86
Elastane 3.5	Weftwise	35.6	−1.6	−34.86
	Warpwise	0.2	−0.4	−6.76
Elastane 4.0	Weftwise	33.4	−1.8	−35.81
	Warpwise	0.8	−0.8	−7.43
PBT/CO (before treatment)	Weftwise	11.6	1.2	-
	Warpwise	1.6	0	-
PBT/CO	Weftwise	45.2	11	−30.95
	Warpwise	5	2	−8
PBT Filament (before treatment)	Weftwise	37	0.6	-
	Warpwise	1.6	0	-
PBT Filament	Weftwise	94.2	9.6	−35.9
	Warpwise	3	0	−6.57

**Table A3:** The air permeability, the thermal comfort, weight, and thickness values of the fabric samples.

Designation	Weight (g/m^2^)	Thickness (mm)	Air Permeability (l/m^2^/s)	Thermal Conductivity (W/mK)	Thermal Absorptivity (Ws^1/2^/m^2^ K)
100% Cotton	267.2	1.326	303.8	0.0613	149.92
PBT/CO	337.8	1.446	99.2	0.0675	163.90
PBT Filament	225.7	1.061	118.4	0.0512	133.70
Elastane 3.0	441.7	1.705	19.5	0.0742	191.92
Elastane 3.5	460.3	1.797	19.2	0.0710	191.52
Elastane 4.0	456.2	1.661	22.02	0.0736	199.14

**Table A4:** Selected physical properties of samples before treatment (b.t.) and after treatment (a.t.).

Designation	Mass Per Unit Area b.t.	Mass Per Unit Area a.t.	Thickness b.t	Thickness a.t.	Bulk Density b.t.	Bulk Density a.t.
	(g/m^2^)	(g/m^2^)	(mm)	(mm)	(g/m^3^)	(g/m^3^)
100% cotton	224.20	277.96	0.826	1.111	0.271	0.250
Cotton/PBT core	162.72	183.97	0.73	0.906	0.223	0.203
PBT filament	182.35	226.82	1.103	1.179	0.165	0.192
Lycra 3	304.12	406.91	1.3	1.447	0.234	0.281
Lycra 3.5	313.79	411.90	1.296	1.501	0.242	0.274
Lycra 4	309.02	402.09	1.29	1.464	0.24	0.275

**Table A5:** Selected UV properties of samples before treatment (b.t.) and after treatment (a.t.).

Designation	UPF b.t.	UPF a.t.	T b.t.	T a.t.	R b.t.	R a.t.	A b.t.	A a.t.
			(%)	(%)	(%)	(%)	(%)	(%)
100% cotton	33.00	59.66	4.59	3.18	36.44	34.83	58.97	61.99
Cotton/PBT core	10.84	17.72	11.65	8.00	35.96	35.94	52.39	56.06
PBT filament	18.46	49.90	9.78	4.91	34.03	36.14	56.19	58.95
Lycra 3	126.00	429.82	1.51	0.58	35.65	38.37	62.84	61.05
Lycra 3.5	157.85	384.30	1.23	0.64	35.18	34.77	63.59	64.59
Lycra 4	150.54	448.36	1.59	0.55	34.36	32.70	64.35	66.75

## Appendix B

**Table A6:** Shrinkage of samples after treatment in warp and weft directions.

Type of PBT	Shrinkage (%)					
	Warp Direction			Weft Direction		
	1 _TWILL_	2 _TWILL_	3 _TWILL_	1 _TWILL_	2 _TWILL_	3 _TWILL_
PBT	3.8	3	4.8	12.7	14.4	26.8
Co/PBT	4.4	4	5.4	11.1	10	16.4
CV/PBT (20 × 2 tex)	4.5	2.7	3.8	16.5	13.2	26
CV/PBT (10 × 2 tex)	4 _PLAIN_	5 _BASKET_	6 _BASKET_	4 _PLAIN_	5 _BASKET_	6 _BASKET_
In every 4th weft	5.0	5.1	7.3	19.5	32.1	44.9
In every 5th weft	5.4	5.7	7.0	15.4	28.2	44.4

**Table A7:** Physical properties of samples before (b.t.) and after (a.t.) treatment.

**Type of PBT**	**Warp Density** **(ends/cm)**					
	**b.t.**			**a.t.**		
	**1 _TWILL_**	**2 _TWILL_**	**3 _TWILL_**	**1 _TWILL_**	**2 _TWILL_**	**3 _TWILL_**
PBT	21.5	20.7	21.6	23.6	23.1	26.6
Co/PBT	21.3	21.3	21.1	23.8	23.7	25.8
CV/PBT (20 × 2 tex)	21.4	21.3	21.3	25.1	21.7	26.8
CV/PBT (10 × 2 tex)	**4 _PLAIN_**	**5 _BASKET_**	**6 _BASKET_**	**4 _PLAIN_**	**5 _BASKET_**	**6 _BASKET_**
Every 4th weft	21.08	21.24	21.56	24.0	29.4	37.76
Every 5th weft	20.88	20.84	21.6	24.14	28.84	37.76
	**Weft Density** **(pics/cm)**					
	**b.t.**			**a.t.**		
	**1 _TWILL_**	**2 _TWILL_**	**3 _TWILL_**	**1 _TWILL_**	**2 _TWILL_**	**3 _TWILL_**
PBT	23.8	28.4	28	25.2	29.4	30.2
Co/PBT	29	29.8	28.9	30.7	30.2	30.5
CV/PBT (20 × 2 tex)	28.8	29.4	29.0	31.5	30.3	31.0
CV/PBT (10 × 2 tex)	**4 _PLAIN_**	**5 _BASKET_**	**6 _BASKET_**	**4 _PLAIN_**	**5 _BASKET_**	**6 _BASKET_**
Every 4th weft	22.04	21.92	21.72	22.6	22.9	24.0
Every 5th weft	22.16	21.68	21.44	23.04	22.72	23.5
	**Thickness** **(mm)**					
	**b.t.**			**a.t.**		
	**1 _TWILL_**	**2 _TWILL_**	**3 _TWILL_**	**1 _TWILL_**	**2 _TWILL_**	**3 _TWILL_**
PBT	0.29	0.34	0.36	0.48	0.57	0.85
Co/PBT	0.30	0.36	0.39	0.48	0.52	0.72
CV/PBT (20 × 2 tex)	0.33	0.43	0.39	0.54	0.58	0.90
CV/PBT (10 × 2 tex)	**4 _PLAIN_**	**5 _BASKET_**	**6 _BASKET_**	**4 _PLAIN_**	**5 _BASKET_**	**6 _BASKET_**
Every 4th weft	0.18	0.21	0.27	0.33	0.64	1.10
Every 5th weft	0.17	0.20	0.25	0.30	0.63	1.12
	**Mass Per Unit Area** **(g/m^2^)**					
	**b.t.**			**a.t.**		
	**1 _TWILL_**	**2 _TWILL_**	**3 _TWILL_**	**1 _TWILL_**	**2 _TWILL_**	**3 _TWILL_**
PBT	76.43	75.29	75.08	87.16	90.58	104.26
Co/PBT	85.13	88.99	85.56	102.5	102.74	107.42
CV/PBT (20 × 2 tex)	95.76	96.11	94.67	115.91	112.89	134.13
CV/PBT (10 × 2 tex)	**4 _PLAIN_**	**5 _BASKET_**	**6 _BASKET_**	**4 _PLAIN_**	**5 _BASKET_**	**6 _BASKET_**
Every 4th weft	81.1	78.6	82.4	102.1	118.3	167.1
Every 5th weft	78.5	75.8	82.5	93.5	112.0	150.4

## Appendix C

**Table A8:** Designation of the samples.

Sample Designation	Yarn Characteristics in Weft	Three Different Weaves with Different Floating	Treatment	On-Loom Settings
Co/PBT—UN1	Co/PBT core spun yarn 40 tex with PBT core yarn 83 dtex	plain	Fabrics treated in boiling water	Warp—8 × 2 tex Warp density—22 ends/cm Weft density—15 ends/cm
Co/PBT—UN2		Twill 1/3 Z		
Co/PBT—UN3		Twill 1/5 Z		
Co/PBT—TR1	Co/PBT core spun yarn 40 tex with PBT core yarn 83 dtex	plain	Co/PBT yarn treated in boiling water before weaving	
Co/PBT—TR2		Twill 1/3 Z		
Co/PBT—TR3		Twill 1/5 Z		

**Table A9:** Physical properties of samples before (b.t.) and after (a.t.) treatment.

Sample Designation	Thickness (mm)		Mass Per Unit Area (g/m^2^)		Warp/Weft Density (Yarns/10 cm)		Dimensional Changes (Shrinkage) of Fabrics after Treatment (%)	
	b.t.	a.t.	b.t.	a.t.	b.t.	a.t.	Warpwise	Weftwise
Co/PBT—UN1	0.35	0.61	110.75	124.14	208/154	248/170	−12.8	−18.6
Co/PBT—UN2	0.47	0.93	117.75	151.46	210/152	290/166	−11.1	−34.15
Co/PBT—UN3	0.46	1.24	118.00	196.2	210/152	369/164	−10.8	−46.45
Co/PBT—TR1	0.39	/	137.1	/	212/148	/	/	/
Co/PBT—TR2	0.53	/	145.5	/	220/150	/	/	/
Co/PBT—TR3	0.58	/	145.4	/	220/150	/	/	/

## References

[1] Rahman M.A. (2011). Effect of Spandex Ratio on the Properties of Woven Fabrics Made of Cotton/Spandex Spun Yarns. J. Am. Sci..

[2] Varghese N., Thilagavathi G. (2014). Development of woven stretch fabrics and analysis on handle, stretch, and pressure comfort. J. Text. Inst..

[3] Mittmann J., Ott R. (1999). Effect of the fabric construction on the elastic properties of woven fabrics containing elastane yarns. Melliand Int..

[4] Luo J., Wang F.-M., Li D., Xu B. (2011). Elasticity of woven fabrics made of polytri-methylene terephthalate/polyethylene terephthalate bicomponent filaments. Text. Res. J..

[5] Dimitrovski K. Structural functionalisation of woven fabrics. Proceedings of the ISF2014.

[6] Štrukelj D., Dimitrovski K. (2012). Study of cotton woven fabrics with added polybutylene terephthalate yarns. Tekstil.

[7] Rijavec T., Bukošek V. (2009). Novel Fibres for the 21st Century. Tekstilec.

[8] Kawaguchi A., Murakami S., Fujiwara M., Nishikawa Y. (2000). Dynamical observation of structural transition of polymers using an X-ray diffraction system with imaging plates. II. Crystalline transition of poly(butylene terephthalate). J. Polym. Sci.

[9] Yokouchi M., Sakakibara Y., Chatani Y., Tadokoro H., Tanaka T., Yoda K. (1976). Structures of Two Crystalline Forms of Poly(butylene terephthalate) and Reversible Transition between Them by Mechanical Deformation. Macromolecules.

[10] Verdu P., Rego J.M., Nieto J., Blanes M. (2009). Comfort Analysis of Woven Cotton/Polyester Fabrics Modified with a New Elastic Fiber, Part 1 Preliminary Analysis of Comfort and Mechanical Properties. Text. Res. J..

[11] Shi X.Q., Ito H., Kikutani T. (2006). Structure development and properties of high-speed melt spun poly (butylene terephthalate)/poly(butylene adipate-co-terephtalate) bicomponent fibres. Polymer.

[12] Chen Q., Ma P., Mao H., Miao X., Jiang G. (2017). The effect of knitting parameter and finishing on elastic property of PET/PBT warp knitted fabric. Autex Res. J..

[13] Zhao L., Hu H., Shey J., Rong H. (2013). The use of polytrimethylene terephtalate/polyester bi-component filament fort he development of seamless garment. Text. Res. J..

[14] Bizjak M., Kadoğlu H., Kostajnšek K., Çelik P., Başal Bayraktar B., Duran D., Bedez Üte T., Ertekin M., Dimitrovski K. Properties of elastic fabrics with treated and untreated Co/PBT yarns in weft direction. Proceedings of the V: AUTEX 2017 World Textile Conference.

[15] Dimitrovski K., Kadoğlu H., Kostajnšek K., Çelik P., Başal Bayraktar G., Duran D., Bedez Üte T., Ertekin M., Bizjak M. From research to implemented product, XIVth International Izmir Textile and Apparel Symposium, Izmir, 2017. Proceedings of the 14th IITAS 2017.

[16] Kadoğlu H., Dimitrovski K., Marmali A., Çelik P., Başal Bayraktar G., Bedez Ü.T., Ertekin G., Demšar A., Kostajnšek K. (2016). Investigation of the Characteristics of Elasticised Woven Fabric by Using PBT Filament Yarns. Autex Res. J..

[17] Dimitrovski K., Kostajnšek K., Gündüz A., Tanyely M., Akleylek A. Properties of cotton-like woven fabrics containing different types pf PBT yarns in the weft. Proceedings of the 6th ITC&DC.

[18] Hümeyra S., Aydoğdu Ç., Yilmaz D. (2020). Effect of yarn fineness and core/sheath fibre types on the physical properties of dual-core yarns and fabrics. Cellul. Chem. Technol..

[19] Mohd Ishak Z.A., Leong Y.W., Steeg M., Karger-Kocsis J. (2007). Mechanical properties of woven glass fabric reinforced in situ polymerized poly (butylene terephthalate) composites. Compos. Sci. Technol..

[20] Baets J., Godara A., Devaux J., Verpoest I. (2008). Toughening of polymerized cyclic butylene terephthalate with carbon nanotubes for use in composites. Compos. Part A Appl. Sci. Manuf..

[21] Metanawin T., Jamjumrus A., Metanawin S. Morphology, Mechanical and Thermal Properties of PBT-TiO_2_ Polymer Nanocomposite, MATEC Web of Conference. Proceedings of the 4th International Conference on Material Science and Engineering Technology (ICMSET 2015).

[22] Hu C.C., Chang S.S., Liang N.Y. (2016). Preparation and characterization of carbon black/polybutylene terephthalate/polyethylene terephthalate antistatic fiber with sheath–core structure. J. Text. Inst..

[23] Gérard E., Bessy E., Salvagnini C., Rerat V., Momtaz M., Hénard G., Marmey P., Verpoort T., Marchand-Brynaert J. (2011). Surface modifications of polypropylene membranes used for blood filtration. Polymer.

[24] Cao Y., Liu J., Zhong R., Yu Q., Wang H. (2012). Surface modification of PBT nonwoven fabrics used for blood filtration and their blood compatibility study. Artif. Cell Blood Sub..

[25] Cao Y., Wang H., Yang C., Zhong R., Lei Y., Sun K., Liu J. (2011). In vitro studies of PBT Nonwoven Fabrics adsorbent for the removal of low density lipoprotein from hyperlipemia plasma. Appl. Surf. Sci.

[26] Miot S., Scandiucci de Freitas P., Wirz D., Daniels A.U., Sims T.J., Hollander A.P., Mainil-Varlet P., Heberer M., Martin I. (2006). Cartilage Tissue Engineering by Expanded Goat Articular Chondrocytes. J. Orthop..

[27] Çelik P., Bedez Ü.T., Kadoğlu H., Marmaralı A., Ertekin G., Kostajnšek K., Demšar A., Dimitrovski K. Comparative study of UV properties of cotton woven fabrics containing lycra and PBT. Proceedings of the 7th ITC&DC.

[28] Arahne. https://www.arahne.si/sl/.

[29] Urbas R., Kostajnšek K., Dimitrovski K. (2011). Impact of structure and yarn colour on UV properties and air permeability of multilayer cotton woven fabrics. Text. Res. J..

[30] Stanković S.B., Popović D., Poparić G.B., Bizjak M. (2009). Ultraviolet protection factor of gray-state plain cotton knitted fabrics. Text Res. J..

